# Genetic Predictors of Early-Onset Spinal Intervertebral Disc Degeneration: Part Two of Two

**DOI:** 10.7759/cureus.15183

**Published:** 2021-05-22

**Authors:** Brian Fiani, Claudia Covarrubias, Ryan Jarrah

**Affiliations:** 1 Neurosurgery, Desert Regional Medical Center, Palm Springs, USA; 2 School of Medicine, Universidad Anáhuac Querétaro, Santiago de Querétaro, MEX; 3 College of Arts and Sciences, University of Michigan-Flint, Flint, USA

**Keywords:** asporin, cartilage intermediate layer protein, insulin like growth factor 1 receptor, matrix metallopeptidase 9, thrombospondin 2, intervertebral disc cells, degenerative spine disease, predictive genetic testing, genetic targets

## Abstract

Understanding genetic indicators is a fundamental aspect to characterizing the pathophysiology of chronic diseases such as intervertebral disc degeneration (IVDD). In our previous spinal genetics review, we characterized some more common genetic influencers in the context of IVDD. In this second part of our two-part comprehensive spinal genetics review, we characterize the more infrequently studied genes that have pathophysiological relevance. In doing so, we aim to expand upon the current gene-library for IVDD. The genes of interest include: *asporin*, *cartilage intermediate layer protein*, *insulin-like growth factor 1 receptor*, *matrix metallopeptidase 9*, and *thrombospondin 2*. Findings show that these genetic indicators have trends and polymorphisms that may have causal associations with the manifestation of IVDD. However, there is a narrow selection of studies that use genetic indicators to describe correlations to the severity and longevity of the pathology. Nevertheless, with the continued identification of risk genes involved with IVDD, the possibilities for refined models of gene therapies can be established for future treatment trials.

## Introduction and background

Intervertebral disc degeneration (IVDD) is a common condition in which the connective disc between the vertebrae undergoes gradual decay over time [1]. Despite being described as a natural part of the aging process, its can happen in patients as early as 11 years of age [1]. Moreover, its symptomatology of chronic back and neck pain makes this condition one of the most debilitating clinical syndromes, with significant physical, psychological, and socioeconomic impacts [1]. In part one of our two-part comprehensive spinal genetics review, we recognized the complexity and ambiguity of IVDD’s pathophysiology. We mentioned how IVDD is a multifactorial condition that can originate from mechanical, biochemical, or environmental stressors. However, its genetic influence has now shown predominance [2].

The increased application of genome and exome sequencing has facilitated a new era of understanding painful and progressive pathologies [3]. The genetic component to polygenic pathologies such as IVDD has been increasingly more characterized, with genome-wide association studies finding mutants, variants, and polymorphisms to genes that adversely influence the structure and function of the intervertebral disc (IVD) [4]. These genes may either gain or lose their function in supporting the IVD, leading to degenerative, apoptotic, and inflammatory signaling pathways that stimulate the pathogenesis of IVD degeneration [5]. However, as the genome library of IVDD expands, more uncommon genes begin to emerge that could have a role in supporting the structure of the IVD disc, activating enzymes that promote the extracellular matrix (ECM), or promoting connective tissue formation. Therefore, mutations to these genes may also promote the pathogenesis of IVDD. In this second part of our two-part series describing the genetic influences of IVDD, we characterize the role of the genes that are comparatively less studied in the context of IVDD.

## Review

Asporin

*Asporin* (*ASPN*) is a gene located on chromosome 9q22.31 encoding for an ECM protein belonging to a small leucine-rich proteoglycan (SLRP) family [6-8]. The encoded protein binds collagen and calcium, thus inducing collagen mineralization [8]. Additionally, ASPN regulates chondrogenesis by inhibiting transforming growth factor-β (TGF-β) gene expression that is commonly expressed in cartilaginous tissues [8,9]. *ASPN* contains a specific polymorphic D-repeat in the N-terminus region, ranging from 9-20 residues [6]. There is an established association between the various D polymorphisms and increased risk of osteoarthritis, making this gene a possible candidate as a susceptibility gene to lumbar disc degeneration (LDD) [6,8,9]. A meta-analysis published in 2008 determined that the *ASPN* gene was shown to be upregulated with age and disc degeneration. Additionally, the reported findings indicated an association between the presence of at least one D14 allele and lumbar disc herniation or degenerative disc disease (DDD) [7].

**Table 1:** Characteristics of less-studied genetic factors associated with disc degeneration. ^a^Systematic review and meta-analysis; ^b^Only two out of a total of 74 studies pertained to MMP9. *ASPN*: asporin; *CILP*: cartilage intermediate layer protein; IDD: intervertebral disc degeneration; *IGF1R*: Insulin-like growth factor 1 receptor; LDD: lumbar disc degeneration; *MMP9*: matrix metallopeptidase 9; *THBS2*: thrombospondin 2

Gene name	Genomic region	Encoded protein family	Selected studies	Number of studies included^a^	Countries or ethnicities included	Findings
ASPN	9q22.31	SLRP family	Song et al., 2008 [6]^a^	2	China, Japan	There was a trend for an association between *ASPN* and severity of LDD in the Chinese cohort.
CILP	15q22.31	Glycoprotein	Wang et al., 2016 [10]^a^	5	Japan, China, Finland	The allele T of rs2073711 of *CILP* is a high-risk allele for IVD.
IGF1R	15q26.3	Anabolic growth factor	Liu et al., 2015 [11]	N/A	China	*IGF1* levels in LDD patients were significantly lower than non-LDD subjects. Significant deactivation of IGF1R in LDD discs when compared to non-LDD subjects was detected.
MMP9	20q13.12	MMP family	Rigal et al., 2017 [12]^a^	2 (74)^b^	Japan, Finland	*MMP-9* rs17576 increases the risk of disc degeneration.
THBS2	6q27	Thrombospondin family	Yuan et al., 2018 [13]	N/A	China	The 2 SNPs (rs6422747 and rs6422748) in the *THBS2* gene were associated with susceptibility of IDD but not severity.
			Deguchi et al., 2019 [14]	N/A	Japan	*THBS2* rs9406328 is associated with IDD. The highest odds ratio was found for rs9406328 in the *THBS2* gene at disc level T12-L1.

Cartilage intermediate layer protein

*Cartilage intermediate layer protein* (*CILP*) gene is located in chromosome 15q22.31 and encodes the CILP glycoprotein [10]. *CILP* is almost exclusive to cartilage tissues such as those found in the IVD [15,16]. This protein is known to accelerate the disc degeneration process by altering the IVD ECM metabolism as some functional studies have suggested that it is capable of antagonizing the ligand-induced insulin-like growth factor 1 (IGF1) autophosphorylation and TGF-β1 signaling functions [10,16]. *CILP* expression in IVD tissue increases with overall aging and generative processes, and it is known that overexpression of *CILP* gene in the NP of transgenic mice is associated with LDD [15]. Furthermore, more studies on mice have documented a significant decrease in IVD signal intensity using magnetic resonance imaging (MRI) techniques which represents a measurable early sign of DDD [15,17,18]. He et al. investigated the response of *CILP* expression on IVD tissue in response to mechanical load where there was a noted decreased expression under tensile loading and an increased expression under compressive loading, respectively [18]. These results suggest that the expression and regulation of *CILP* can be dependent on mechanical loading, where high levels of expression in IVDD can be attributed to overloading of the IVD [18,19]. Lastly, the first meta-analysis investigating the relationship between IVD and *CILP* , which included both European and Asian populations, was published in 2016. Using a total of five cohorts and 3,344 study subjects, the authors determined that the allele T of rs2073711 of *CILP* gene was a high-risk allele for IVD [10]. Due to the limited data and lack of diversity in the populations studied in association with IVD and *CILP* polymorphisms, more studies need to be conducted and expanded to other ethnic groups as well as appending gender-specific association analyses.

Insulin-like growth factor 1 receptor

The *insulin-like growth factor 1 receptor* (*IGF1R*) encodes for a receptor that binds insulin-like growth factor (IGF) with a high affinity. IGF1, also called somatomedin C, is an anabolic polypeptide hormone that plays a major role in tissue growth and development and is known to stimulate ECM synthesis [20,21]. Some rodent studies have proposed IGF1 as a therapeutic strategy in the presence of both IVDD and type 2 diabetes mellitus. As previously mentioned, IGF1 is known to directly promote chondrocyte proliferation, and ACAN synthesis in the NP. Additionally, it acts as a glucose suppressor and can aid in maintaining the expression of other ECM proteins, such as collagen and ACAN, as these can be inhibited in the presence of hyperglycemia [20,21]. The IGF1R is a tyrosine kinase receptor that activates phosphatidylinositol-3 kinase (PI3k)/Akt signaling pathway, a natural stimulator of cell proliferation and growth and apoptosis inhibitor [13]. Liu et al. analyzed the effects of IGF1 on the activation of *IGF1R*, Akt, and *MMP3* in human NP where they identified that LDD patients had lower serum and IGF1 levels when compared to non-LDD subjects [11].

Matrix metallopeptidase 9

*Matrix metallopeptidase 9* (*MMP9*), like *MMP2*, is a metalloproteinase gene encoding an enzyme from the zinc-dependent proteinase family [22]. *MMP9 *is subcategorized as a gelatinase that digests denatured gelatins, collagens, and laminin. *MMPs *are traditionally known as the primary mediators of ECM degradation likely involved in normal tissue repair and remodeling. Dysregulation and aberrant expression of these enzymes are believed to be responsible for the propagation of diseases such as osteoarthritis and IVDD [22]. A 1562C/T polymorphism in the *MMP9* promoter region was studied in Chinese males with LDD where there was an association between the CC/TT *MMP9* genotype in relation to MRI evidence of severe IDD [23]. Additionally, the *MMP9* rs17576 (GG) genotype was recently studied through a meta-analysis analyzing two articles that concluded a positive association between the rs17576 polymorphism and increased risk of DDD [12].

Thrombospondin 2

*Thrombospondin 2*(*THBS2*) gene encodes the thrombospondin 2 protein, a glycoprotein, that regulates the effective levels of catabolic proteins, such as *MMP2 *and *MMP9*, present in the ECM [2]. *THBS2 *mediates cell-to-cell and cell-to-matrix interactions during tissue repair and development and has also been shown to function as an angiogenic inhibitor [13,24]. The allelic variation rs9406328 has been reported to have an effect on *THBS2 *binding with proteins leading to the activation of MMPs [7]. A study in China associated two SNPs in the *THBS2 *gene, rs6422747 and rs6422748, with IDD susceptibility [13]. Furthermore, Deguchi et al. associated *THBS2* rs9406328 with IDD, as well as significant interaction between *THBS2* and age to IDD was detected in the thoracolumbar junction and thoracic spines [14]. Lastly, no concrete conclusions can be made regarding the role of *THBS2* and IDD as there is no meta-analysis on the subject to date.

## Conclusions

The genome library of IVDD is expanding. In this second part of our two-part series, we identify and discuss the literature to date for the uncommonly studied genes with the goal of providing a more comprehensive understanding of the genetic profile of IVD. Nevertheless, there is a paucity of studies that stratify and compare these genes based on patients with varying clinical courses and their symptomatology. By finding patterns and quantifying gene expression among varying patient disc samples, clinicians can develop genetic markers that can better predict the pathogenesis of IVDD. Further studies are warranted to better address the genetic influence of IVDD to refine future therapies and augment the effort to improve the outcomes of patients at risk. Such studies would allow for more sophisticated therapies that can target mutated genes at the level of expression.
